# Progesterone impairs antigen-non-specific immune protection by CD8 T memory cells via interferon-γ gene hypermethylation

**DOI:** 10.1371/journal.ppat.1006736

**Published:** 2017-11-20

**Authors:** Yushi Yao, Hui Li, Jie Ding, Yixin Xia, Lei Wang

**Affiliations:** 1 McMaster Immunology Research Center, Michael G. DeGroote Institute for Infectious Disease Research, McMaster University, Hamilton, Ontario, Canada; 2 Department of Hematology, Chinese PLA General Hospital, Beijing, China; 3 Department of Clinical Nutrition, General Hospital of Chinese People's Armed Police Forces, Beijing, China; 4 Department of Obstetrics and Gynecology, General Hospital of Chinese People's Armed Police Forces, Beijing, China; National Jewish Health, UNITED STATES

## Abstract

Pregnant women and animals have increased susceptibility to a variety of intracellular pathogens including *Listeria monocytogenes* (LM), which has been associated with significantly increased level of sex hormones such as progesterone. CD8 T memory(Tm) cell-mediated antigen-non-specific IFN-γ responses are critically required in the host defense against LM. However, whether and how increased progesterone during pregnancy modulates CD8 Tm cell-mediated antigen-non-specific IFN-γ production and immune protection against LM remain poorly understood. Here we show in pregnant women that increased serum progesterone levels are associated with DNA hypermethylation of IFN-γ gene promoter region and decreased IFN-γ production in CD8 Tm cells upon antigen-non-specific stimulation *ex vivo*. Moreover, IFN-γ gene hypermethylation and significantly reduced IFN-γ production post LM infection in antigen-non-specific CD8 Tm cells are also observed in pregnant mice or progesterone treated non-pregnant female mice, which is a reversible phenotype following demethylation treatment. Importantly, antigen-non-specific CD8 Tm cells from progesterone treated mice have impaired anti-LM protection when adoptive transferred in either pregnant wild type mice or IFN-γ-deficient mice, and demethylation treatment rescues the adoptive protection of such CD8 Tm cells. These data demonstrate that increased progesterone impairs immune protective functions of antigen-non-specific CD8 Tm cells via inducing IFN-γ gene hypermethylation. Our findings thus provide insights into a new mechanism through which increased female sex hormone regulate CD8 Tm cell functions during pregnancy.

## Author summary

Increased female sex hormones during pregnancy generate a temporary immune suppression status in the pregnant that protect the developing fetus from maternal rejection but renders the pregnant highly susceptible to various pathogens. However, molecular mechanisms underlying such an increased maternal susceptibility to pathogens during pregnancy remain to be further understood. Here we show in pregnant women that increased progesterone levels are associated with IFN-γ gene hypermethylation and reduced IFN-γ production in peripheral CD8 Tm cells. By using murine models of LM infection, for the first time we show a causal relationship between increased level of progesterone, a characteristic female sex hormone of pregnancy, and increased susceptibility to *Listeria monocytogenes*, an intracellular bacterium that endangers both the pregnant and the fetus. Such an impact on anti-listeria host defense is mediated through progesterone-induced IFN-γ gene hypermethylation in CD8 Tm cells, resulting in impaired IFN-γ production and reduced immune protection by antigen-non-specific CD8 Tm cells. This study provides new insights into molecular mechanisms underlying the increased susceptibility to intracellular pathogens during pregnancy.

## Introduction

Increased susceptibility to a variety of pathogens during pregnancy has been related to a temporary status of immune suppression induced by increased female sex hormones such as progesterone and estrogen[1–5]. Indeed, previous studies showed that female sex hormones play regulatory roles in various human immune cells *ex vivo*[6,7]. In animal models, progesterone and estrogen have been shown to exert immune regulatory roles that facilitate maternal-fetal tolerance and protect animals from autoimmune diseases such as experimental autoimmune encephalomyelitis[7–10]. It has also been shown in animal models of infections including influenza virus infections that progesterone reduces anti-virus cellular immune responses while at the same time limits immunopathology[11–14]. Despite of these understandings, roles of female sex hormone in increased susceptibility to infections during pregnancy and the underlying cellular and molecular mechanisms remain to be further defined[3,4,7].

Pregnant women and animals are at higher risks of infection with *Listeria monocytogenes*(LM), an Gram positive intracellular bacterium[15–19]. In Europe, the incidence rate of listeriosis was estimated to vary between 0.1 and 11.3 per million population, with approximately 20% neonatal infections[20]. In the USA, there were 758 reported cases of listeriosis during 2004–2007, with 16.9% pregnant associated[21]. Although mostly asymptomatic, LM infection during pregnancy can be dangerous not only to the maternal body but also fatal to the developing fetus[17,18]. Innate immunity is critical to optimal control of LM[15,22–25]. Early studies carried out in mouse models showed that IFN-γ produced by NK cells triggered by IL-12 and IL-18 activates bactericidal functions of macrophages against phagocytized LM and is thus critically required in innate bacterial control early after infection[22–24]. Moreover, innate immune responses against LM are important to the establishment of subsequent adaptive immune responses and facilitate bacterial clearance by T cells[15,25]. Conventionally, innate immune responses against LM are restricted to innate immune cells such as NK cells and macrophages[15]. However, recent studies showed that CD8 T memory(Tm) cells provide antigen-non-specific and innate-like immune protection early after LM infection via IFN-γ production in a cognate antigen-independent but innate cytokine dependent manner[26,27]. Notably, such an antigen-non-specific immune protection by CD8 Tm cells is more prominent than that of NK cells due to preferential co-localization of CD8 Tm cells with LM and macrophages[27]. Thus, IFN-γ produced by antigen-non-specific CD8 Tm cells is indispensable to host defense against LM.

The risk of LM infection in pregnant women is highest in the third trimester of pregnancy, when maternal serum progesterone levels are 5–10 folds higher than that before pregnancy[4,28]. Indeed, it has been reported that progesterone modulates functions of various immune cells, regardless of known progesterone receptor expression in these cells[7]. In human CD8 T cells, progesterone has been shown to reduce IFN-γ production upon stimulation *ex vivo*, although the underlying mechanisms remain unknown[6]. IFN-γ production by CD8 T cells is tightly regulated by a series of consequential epigenetic modulating mechanisms[29–33]. It has been shown in CD8 Tm cells that DNA methylation at the CpG sites of IFN-γ gene promoter is a key mechanism through which IFN-γ production by CD8 Tm cells is regulated[33]. Upon recognition of cognate antigens via TCR, CD8 Tm cells have rapidly IFN-γ gene demethylation, facilitating the transcription of IFN-γ gene[31]. Despite of these intriguing findings, it remains unknown whether and how increased progesterone during pregnancy inhibits IFN-γ production by antigen-non-specific CD8 Tm cells that is crucial to host defense against LM.

Here we show in pregnant women that increased serum progesterone levels are associated with DNA hypermethylation of IFN-γ gene and decreased IFN-γ production in CD8 Tm cells upon antigen-non-specific stimulation. In both pregnant mice and progesterone treated non-pregnant female mice, hypermethylation of IFN-γ gene and significantly reduced IFN-γ production by antigen-non-specific CD8 Tm cells upon LM infection are also observed. And such a reduction in IFN-γ production by CD8 Tm cells is reversed following treatment with demethylating agent. More importantly, antigen-non-specific CD8 Tm cells from progesterone treated mice have impaired protection against LM when adoptive transferred in pregnant mice or IFN-γ-deficient mice, and demethylating agent rescues the impaired adoptive protection of antigen-non-specific CD8 Tm cells induced by progesterone. These data demonstrate that increased levels of progesterone impair immune protection of antigen-non-specific CD8 Tm cells against LM, via inducing DNA hypermethylation of IFN-γ gene. Our findings thus reveal a novel mechanism through which increased female sex hormone regulates CD8 Tm cell functions that result in increased susceptibility to intracellular pathogens during pregnancy.

## Results

### Increased serum progesterone level is associated with DNA hypermethylation of IFN-γ gene in human CD8 Tm cells

**Fig 1:**
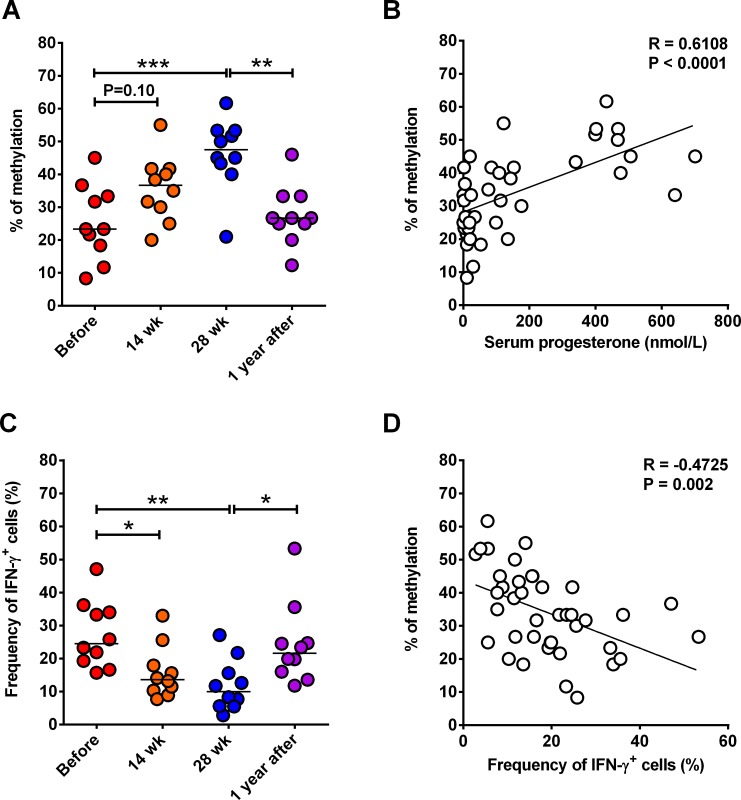
Correlation between progesterone or IFN-γ production with IFN-γ gene methylation in human CD8 Tm cells. CD8 Tm cells were purified from PBMCs of 10 subjects at before, weeks 14 and 28 of pregnancy and around 1 year after delivery. Average percentages of DNA methylation at 6 CpG sites of IFN-γ gene promoter region are shown in (**A**). (**B**) Correlation between serum progesterone levels and IFN-γ gene methylation levels of all samples as shown in (A). Frequencies of IFN-γ-producing PBMC CD8 Tm cells after *ex vivo* stimulation with PMA and Ionomycin are shown in (**C**). And Correlation between IFN-γ gene methylation levels and frequencies of IFN-γ-producing CD8 Tm cells is shown in (**D**). Horizontal lines in (A) and (C) represent median values. One-way ANOVA and Tukey’s multiple comparisons test was used to compare between multiple groups. Pearson correlation analysis was used to determine the potential correlation between two parameters. * P<0.05; ** P<0.01; *** P<0.001. The experiments were performed once.

Pregnant women have significantly reduced host defense against various intracellular pathogens, particularly during the third trimester of pregnancy when serum levels of progesterone reaches the highest levels[18,19,28]. Immune responses mediated by CD8 Tm cells are critically required in host defense against intracellular pathogens[34]. IFN-γ, a key molecule in CD8 T cell functions that is subject to epigenetic regulation pathways including DNA methylation, is reduced by progesterone, a characteristic female sex hormone that is significantly increased during pregnancy[6]. This prompts us to ask whether serum progesterone levels are related to the methylation level at IFN-γ gene promoter region that controls IFN-γ production by CD8 Tm cells from pregnant women. To test this, we purified peripheral blood CD8 Tm cells from 10 women at before, weeks14 and 28 of pregnancy, and approximately 1 year after delivery. Serum progesterone levels were also determined at all the four time points. Methylation levels at six known CpG sites in the promoter region of IFN-γ gene was determined by using bisulfate sequencing. Some CD8 Tm cells were stimulated *ex vivo* with PHA, followed by intracellular staining of IFN-γ. Before pregnancy, median percentage of IFN-γ gene methylation at the six CpG sites was less than 25% (**Fig 1A**). At weeks 14 and 28 of pregnancy, the percentages of IFN-γ gene methylation were around 40% and 50%, respectively, with that of week 28 significantly higher than before pregnancy (**Fig 1A**). One year after delivery, the percentage of IFN-γ gene methylation was reduced to a comparable level to that before pregnancy, being significantly lower than that at week 28 (**Fig 1A**). Correlation analysis data showed that increased serum progesterone level was correlated to hypermethylation of IFN-γ gene promoter CpG sites (**Fig 1B**). Consistent to the IFN-γ gene methylation levels, relative expression of IFN-γ mRNA in CD8 Tm cells upon *ex vivo* stimulation was reduced during pregnancy but not at one year after delivery (**S1 Fig**). And frequency of IFN-γ-producing CD8 Tm cells upon *ex vivo* stimulation was significantly reduced at weeks 14 and 28 of pregnancy as compared to that before pregnancy (**Fig 1C**). One year after delivery, frequency of IFN-γ-producing CD8 Tm cells recovered to a comparable level with that before pregnancy (**Fig 1C**). Not unexpectedly, correlation analysis data showed that frequency of IFN-γ-producing CD8 Tm cells was negatively related to IFN-γ gene methylation levels (**Fig 1D**). Our data thus suggest that increased serum progesterone levels during pregnancy are related to IFN-γ gene hypermethylation and reduced IFN-γ production in CD8 Tm cells.

### Demethylating treatment increases IFN-γ production by CD8 Tm cells from pregnant women

**Fig 2:**
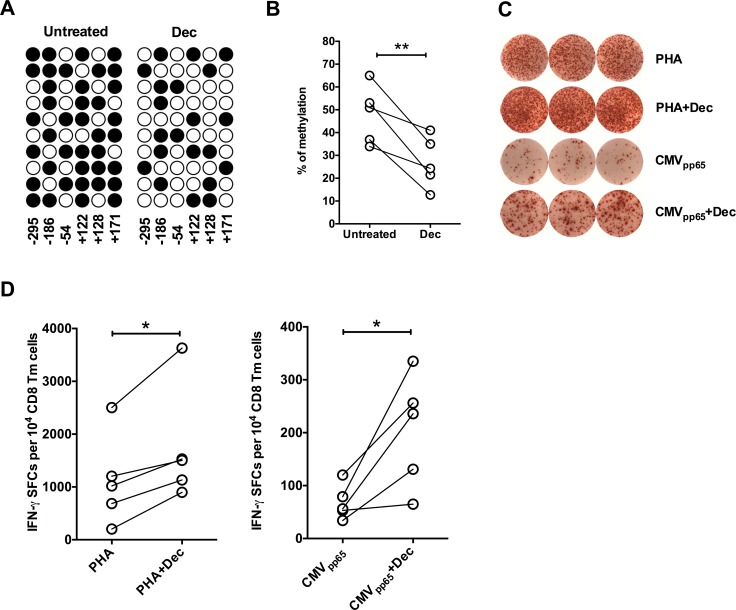
Impact of demethylation treatment on IFN-γ production by CD8 Tm cells from pregnant women. (**A**) Representative distribution of methylation at 6 CpG sites of IFN-γ gene promoter region before and after demethylation treatment (decitabine, Dec) is shown. Numbers refer to position relative to transcription start site. Filled circles represents methylated CpG and open circles represent demethylated CpG. (**B**) Average percentages of DNA methylation at 6 CpG sites of IFN-γ gene promoter region before and after demethylation treatment in PBMC CD8 Tm cells from five women at week 28 of pregnancy. (**C**) Representative graphs of triplicate IFN-γ ELISpot wells of CD8 Tm cells stimulated *ex vivo* with either PHA or T2 cells pulsed with CMV_pp65_ peptide, with or without demethylation treatment. (**D**) Statistics of IFN-γ spot forming CD8 Tm cells from five pregnant women, following *ex vivo* stimulation with PHA or T2 cells pulsed with CMV_pp65_ peptide, with or without demethylation treatment. Two-tailed paired Student’s *t*-test was used for statistical comparison between control and decitabine treatment groups. * P<0.05; ** P<0.01. The experiments were performed once.

To address the causal relationship between IFN-γ gene hypermethylation and reduced IFN-γ production by CD8 Tm cells during pregnancy, we treated CD8 Tm cells from pregnant women at 28 week of pregnancy *ex vivo* with demethylating agent decitabine, followed by stimulation of CD8 Tm cells. Demethylation treatment significantly reduced IFN-γ gene methylation level in CD8 Tm cells from pregnant women at 28 weeks of pregnancy (**Fig 2A and 2B**). Moreover, pre-treatment with demethylating agent significantly increased the frequency of IFN-γ-producing CD8 Tm cells from pregnant women following both TCR-independent stimulation by PHA and TCR-dependent stimulation by CMV_pp65_ peptide *ex vivo* (**Fig 2C and 2D**). Thus our findings suggest that reduced IFN-γ production by CD8 Tm cells during pregnancy is dependent on IFN-γ gene hypermethylation, which is related to increased progesterone level (**Fig 1B**).

### Decreased IFN-γ production by antigen-non-specific CD8 Tm cells in pregnant mice

In pregnant women, there is a large number of circulating CD8 Tm cells that are non-specific for LM antigens[35,36]. And it is speculative, based on murine studies, that LM antigen-non-specific CD8 Tm cells provide immune protection against LM via IFN-γ production[26,27]. To better mimic this immunological scenario in human, we next use a murine model of LM infection during pregnancy to further determine the functional significance of IFN-γ gene hypermethylation and reduced IFN-γ production by antigen-non-specific CD8 Tm cells during pregnancy. We immunized naïve female mice with rAdHuOVA to generate LM non-specific CD8 Tm cells[37]. These immunized female mice were then mated to males to generate pregnant mice with LM non-specific CD8 Tm cells (**S2A and S2B Fig**).

**Fig 3:**
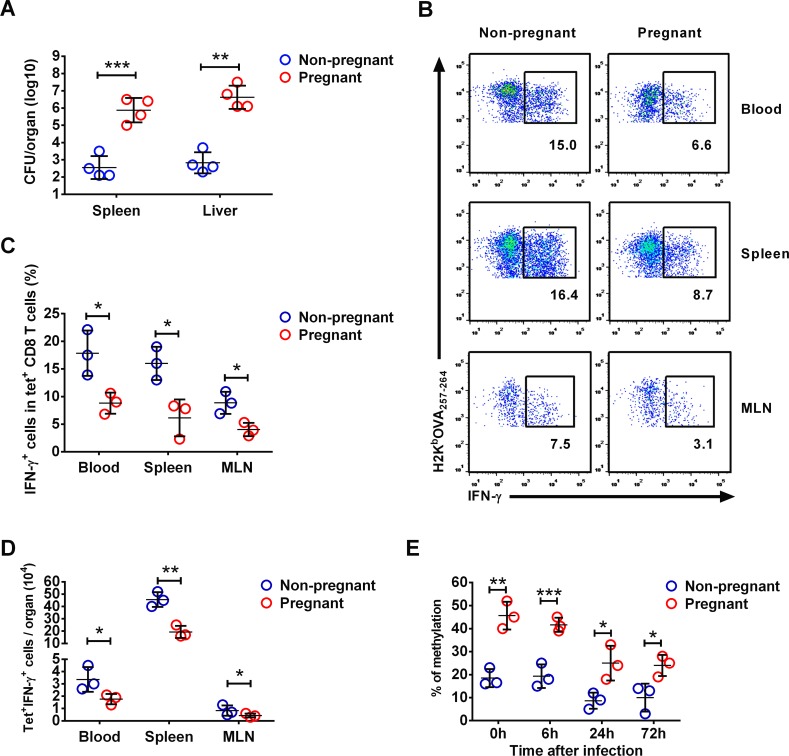
IFN-γ production by antigen-non-specific CD8 Tm cells in pregnant mice. Mice were immunized with rAdHuOVA 40 days before pregnancy, followed by LM infection. Bacterial CFU numbers in the spleen and the liver at 72 hours post infection are shown in (**A**). At 24 hours post infection, OVA antigen-specific CD8 Tm cells (CD8^+^H2K^b^OVA tetramer^+^) from peripheral blood, the spleen and MLN were stained with intracellular IFN-γ. Representative flow cytometry dot plot on intracellular IFN-γ staining in splenic CD8^+^ cells are shown in (**B**). Numbers in dot plots represent percentages of IFN-γ^+^ CD8^+^H2K^b^OVA tetramer^+^ cells in total CD8^+^H2K^b^OVA tetramer^+^ cells. Statistics of frequency (**C**) and absolute number (**D**) of IFN-γ-producing H2K^b^OVA tetramer^+^ CD8 Tm cells in various organs are shown. (**E**) Levels of IFN-γ gene methylation in splenic H2K^b^OVA tetramer^+^ CD8 Tm cells are shown at various time points before and within 72 hours after infection. Error bars represent mean±SD. Two-tailed unpaired Student’s *t*-test was used for statistical comparison between two groups. * P<0.05; ** P<0.01; *** P<0.001. Data in (A) are representatives of 2 independent experiments with n = 4 per group. Data in (B), (C) and (D) are representatives of 2 independent experiments with n = 3 per group. Data in (E) are representatives of 2 independent experiments with n = 3 per group.

Compared to non-pregnant female mice, pregnant mice had over 3 logs higher bacterial CFU number at 72h post LM infection (**Fig 3A**). At 24h post infection, both frequency and absolute number of IFN-γ-producing OVA-specific CD8 Tm cells in peripheral blood, spleen and mesenteric lymph nodes (MLN) were significantly lower in pregnant mice compared to non-pregnant females (**Fig 3B, 3C and 3D**). As these CD8 Tm cells are not LM antigen-specific, our data suggest that IFN-γ production by antigen-non-specific CD8 Tm cells early after LM infection is impaired during pregnancy. We further analyzed IFN-γ gene methylation levels at various time points before and after infection. Although demethylation of IFN-γ gene occurred in both pregnant and non-pregnant mice post infection, the IFN-γ gene methylation level was significantly higher in pregnant mice at all the time points (**Fig 3E and S3A Fig**). Further methylation analysis at distal regulatory elements of IFN-γ gene before LM infection further support a hypermethylation status of IFN-γ gene in pregnant mice (**S3B Fig**). Similar to that observed in pregnant women, our data demonstrate IFN-γ gene hypermethylation and reduced IFN-γ production in antigen-non-specific CD8 Tm cells, which is associated with increased susceptibility to LM in pregnant mice. We also determined NKG2D expression on OVA-specific CD8 Tm cells in various organs in pregnant mice, as NKG2D has been associated with bystander activation of antigen-non-specific CD8 Tm cells. Our data showed that NKG2D expression was not altered in pregnant mice compared to non-pregnant female mice (**S4 Fig**).

### Exogenous progesterone induces DNA hypermethylation of IFN-γ gene in CD8 Tm cells in mice

**Fig 4:**
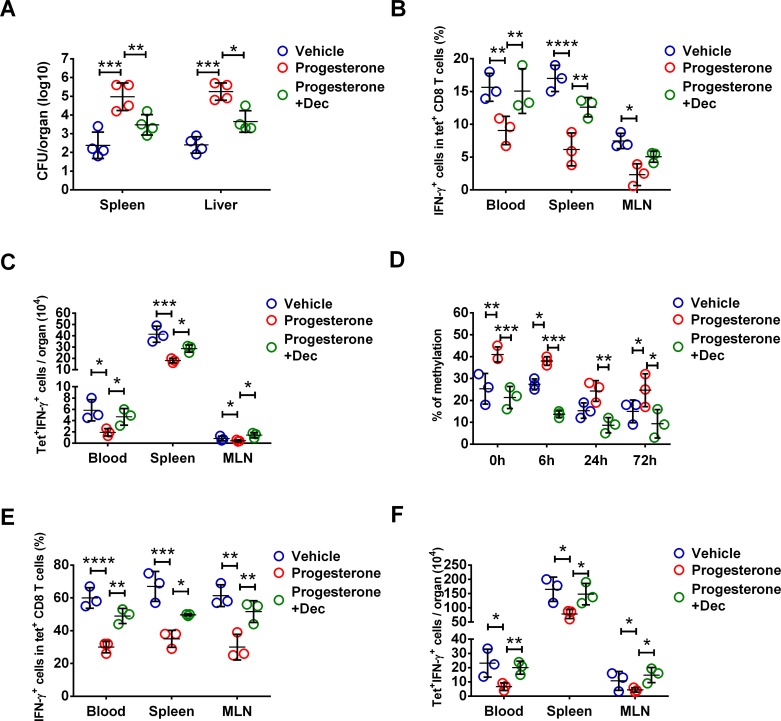
Dependence of DNA hypermethylation in progesterone-induced IFN-γ reduction in antigen-non-specific CD8 Tm cells. Female mice were immunized with rAdHuOVA for 40 days before a 2-week regimen of exogenous progesterone injection, or vehicle as control. Mice were challenged with virulent LM after progesterone administration. In some progesterone injected mice, demethylating agent decitabine (Dec) was administered. Bacterial CFU numbers in the spleen and the liver at 72 hours post infection are shown in (**A**). At 24 hours post infection, OVA antigen-specific CD8 Tm cells (CD8^+^H2K^b^OVA tetramer^+^) from various organs were stained with intracellular IFN-γ. Statistics of frequency (**B**) and absolute number (**C**) of IFN-γ-producing H2K^b^OVA tetramer^+^ CD8 Tm cells in various organs are shown. (**D**) Levels of IFN-γ gene methylation in splenic H2K^b^OVA tetramer^+^ CD8 Tm cells are shown at various time points before and within 72 hours after infection. In (**E**) and (**F**), cells were stimulated *ex vivo* with innate cytokines IL-12 and IL-18. Statistics of frequency (**E**) and absolute number (**F**) of IFN-γ-producing H2K^b^OVA tetramer^+^ CD8 Tm cells in various organs are shown. Error bars represent mean±SD. One-way ANOVA and Tukey’s multiple comparisons test was used to compare between multiple groups. * P<0.05; ** P<0.01; *** P<0.001; **** P<0.0001. Data in (A) are representatives of 2 independent experiments with n = 4 per group. Data in (B) and (C) are representatives of 2 independent experiments with n = 3 per group. Data in (D) are representatives of 2 independent experiments with n = 3 per group. Data in (E) and (F) are representatives of 2 independent experiments with n = 3 per group.

To further determine the impact of increased progesterone levels on IFN-γ gene methylation and IFN-γ production by antigen-non-specific CD8 Tm cells, rAdHuOVA immunized female mice were injected with progesterone for 14 consecutive days. Serum progesterone concentration following such an exogenous progesterone supplementation strategy reflected that in pregnant mice (**S5 Fig**)[38,39]. Some progesterone-treated mice were administered with demethylating agent decitabine. Progesterone treated mice had nearly 3 logs higher bacteria CFU numbers in the spleen and the liver at 72h post LM infection compared to control mice (**Fig 4A**). Demethylation treatment significantly reduced bacteria burden in both the spleen and liver in progesterone treated mice with around 1.5 logs reduction in bacteria CFU numbers (**Fig 4A**). At 24h post infection, both frequency and absolute number of IFN-γ-producing OVA-specific CD8 Tm cells in peripheral blood, spleen and MLN were reduced in progesterone treated mice (**Fig 4B and 4C**). Consistent with the reduced IFN-γ production by OVA-specific CD8 Tm cells, both frequency and absolute number of IFN-γ-producing H2K^b^OVA tetramer^-^ CD8 T cells were reduced in progesterone-treated mice (**S6A and S6B Fig**). These data suggest that progesterone treatment reduces IFN-γ production in both OVA-specific CD8 Tm cells and other CD8 Tm cells such as endogenous antigen-inexperienced CD8 Tm cells[40]. In contrast to CD8 Tm cells, there was a moderate but not significant reduction of IFN-γ-producing NK cells in progesterone-treated mice following LM infection (**S6C and S6D Fig**), suggesting that the impact of progesterone on IFN-γ production is a cell type-specific phenotype. Notably, demethylation treatment significantly increased the frequency and absolute number of IFN-γ-producing OVA-specific CD8 Tm cells in peripheral blood, the spleen and MLN (**Fig 4B and 4C**).

Similar to that in pregnant mice, CD8 Tm cells in progesterone treated mice had higher IFN-γ gene methylation levels at various time points before and after infection (**Fig 4D and S7 Fig**). Such an effect of progesterone on IFN-γ gene methylation was reversed by demethylation treatment (**Fig 4D and S7 Fig**). We also determined IFN-γ production by CD8 Tm cells after *ex vivo* stimulation with innate cytokines IL-12 and IL-18 for 24h without cognate antigen stimulation. As shown in **Fig 4E and 4F**, frequency and absolute number of IFN-γ-producing OVA-specific CD8 Tm cells in peripheral blood, spleen and MLN were reduced in progesterone treated mice. Demethylation treatment rescued the reduced frequency and absolute number of IFN-γ-producing antigen-non-specific CD8 Tm cells induced by progesterone (**Fig 4E and 4F**). These findings demonstrate that progesterone reduces IFN-γ production by antigen-non-specific CD8 Tm cells and impairs host defense against LM via IFN-γ gene hypermethylation.

### Progesterone impairs immune protective functions of antigen-non-specific CD8 Tm cells in pregnant mice

**Fig 5:**
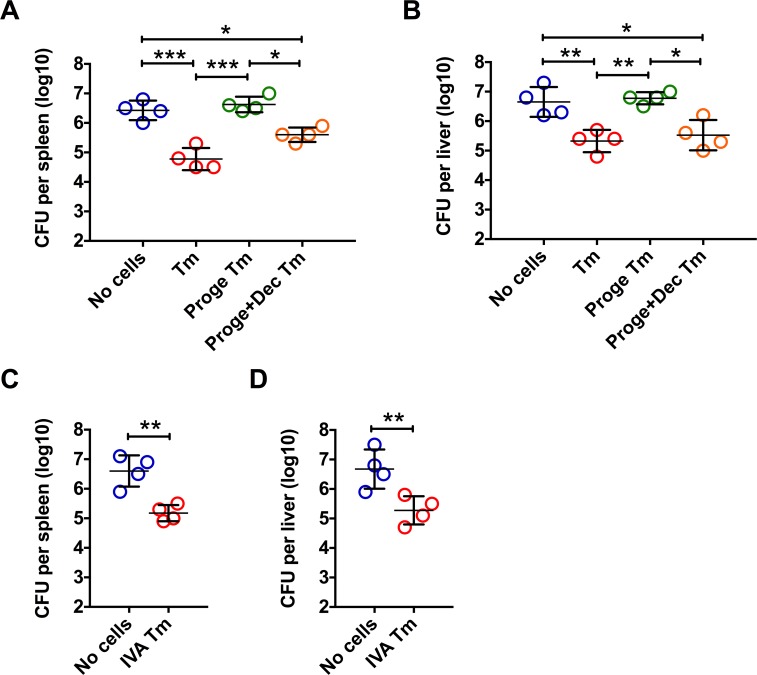
Impact of progesterone on immune protection of antigen-non-specific CD8 Tm cells in pregnant mice. As shown in (**A**) and (**B**), CD8 Tm cells were generated by immunization with rAdHuOVA. At 40 days post immunization, some mice were injected with progesterone(Proge) for 2 weeks, with or without decitabine(Dec) treatment. OVA antigen-specific CD8 Tm cells purified from the spleen and MLN were adoptive transferred into antigen-inexperienced pregnant mice followed by LM infection. Bacterial CFU numbers in the spleen (A) and the liver (B) at 72 hours post infection are shown. In (**C**) and (**D**), influenza A virus NP_366-374_ antigen-specific CD8 Tm cells (IVA Tm) were generated by immunization with the A/PR8/34 influenza virus. At 40 days post immunization, NP_366-374_ antigen-specific CD8 Tm cells purified from the spleen and MLN were pooled and adoptively transferred into antigen-inexperienced pregnant mice followed by LM infection. Bacterial CFU numbers in the spleen (C) and the liver (D) at 72 hours post infection are shown. Error bars represent mean±SD. One-way ANOVA and Tukey’s multiple comparisons test was used to compare between multiple groups. * P<0.05; ** P<0.01; *** P<0.001. Data in (A) and (B) are representatives of 2 independent experiments with n = 4 per group. Data in (C) and (D) are representatives of 2 independent experiments with n = 4 per group.

We next determined whether increased susceptibility of pregnant mice to LM is dependent on progesterone induced impairment of IFN-γ production in antigen-non-specific CD8 Tm cells. To do this, we adoptive transferred OVA-specific CD8 Tm cells or IVA NP_366-374_-specific CD8 Tm cells. As shown in **Fig 5A, 5B, 5C and 5D**, adoptive transfer of either OVA-specific CD8 Tm cells or IVA NP_366-374_-specific CD8 Tm cells significantly reduced LM bacterial burden in LM-naïve host pregnant mice. We also adoptive transferred OVA-specific CD8 Tm cells from mice treated with progesterone alone or in combination with decitabine, into pregnant mice. At 24 hours post LM infection, adoptively transferred OVA-specific CD8 Tm cells from progesterone-treated donor mice had significantly reduced IFN-γ-producing capacity, as compared to those without progesterone treatment or those treated with progesterone and demethylating agent decitabine (**S8A and S8B Fig**). More importantly, adoptive transfer of OVA-specific CD8 Tm cells from progesterone treated mice failed to reduce bacterial burdens in the spleen and liver (**Fig 5A and 5B**). Whereas CD8 Tm cells from either un-treated or progesterone- and decitabine-treated mice significantly reduced bacterial burdens (**Fig 5A and 5B**). These data thus demonstrate that progesterone impairs anti-LM protection by antigen-non-specific CD8 Tm cells via hypermethylation-dependent mechanisms.

As one of the most severe clinical outcomes from gestational LM infection is fetal loss, we also determined pregnancy outcomes in LM-infected pregnant mice following antigen-non-specific CD8 Tm cell transfer. OVA-specific CD8 Tm cells from donor mice without, but not with progesterone treatment, moderately increased number of viable fetus per mouse and moderately decreased abortion rate in LM-infected pregnant mice (**S9A and S9B Fig**), although the differences were not statistically significant.

### Progesterone impairs IFN-γ-mediated protective functions of antigen-non-specific CD8 Tm cells via DNA hypermethylation-dependent mechanism

**Fig 6:**
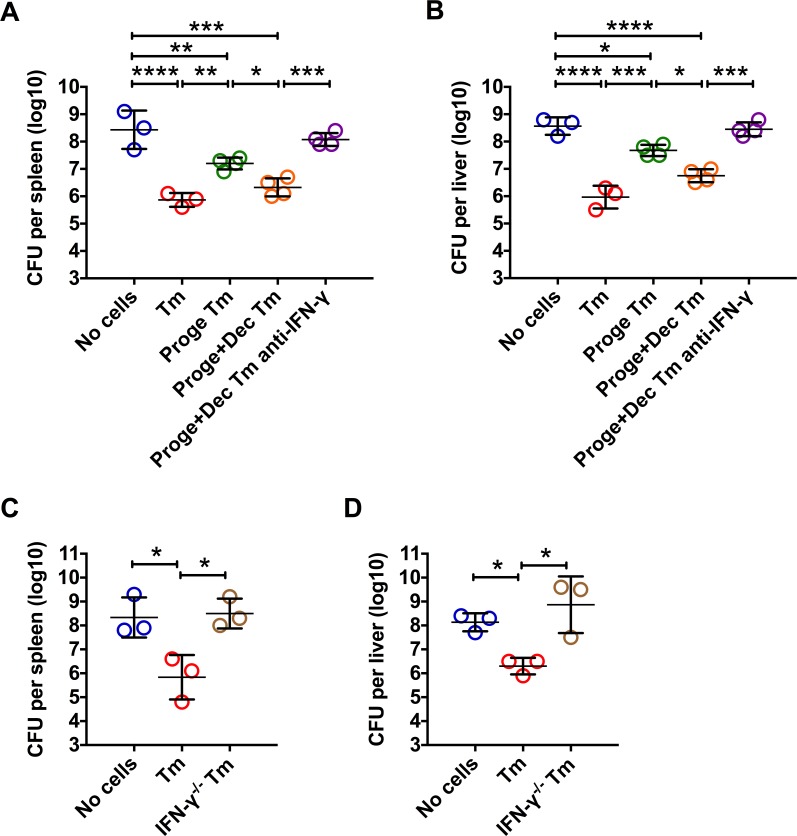
Dependence of IFN-γ in progesterone-induced impairment of protection by antigen-non-specific CD8 Tm cells. CD8 Tm cells were generated by immunization with rAdHuOVA in C57BL/6 mice (Tm) or IFNG-/- mice (IFN-γ^-/-^ Tm). At 40 days post immunization, some immunized C57BL/6 mice were injected with progesterone(Proge) for 2 weeks, with or without decitabine(Dec) treatment. OVA antigen-specific CD8 Tm (Tm or IFN-γ^-/-^ Tm) cells purified from the spleen and MLN were adoptive transferred into naïve IFNG^-/-^ mice followed by LM infection. Some mice adoptive transferred with CD8 Tm cells were treated with anti-IFN-γ neutralizing antibody before and after infection. Bacterial CFU numbers in the spleen (**A** and **C**) and the liver (**B** and **D**) at 72 hours post infection are shown. Error bars represent mean±SD. One-way ANOVA and Tukey’s multiple comparisons test was used to compare between multiple groups. * P<0.05; ** P<0.01; *** P<0.001; **** P<0.0001. Data in (A) and (B) are representatives of 2 independent experiments with n = 3 to 4 per group. Data in (C) and (D) are representatives of 2 independent experiments with n = 3 per group.

To further show that impaired protective functions of antigen-non-specific CD8 Tm cells are dependent on reduced IFN**-γ** production due to DNA hypermethylation, we adoptive transferred OVA-specific CD8 Tm cells from wild type mice treated with progesterone alone or in combination with decitabine, into naïve IFN-γ-deficient **(**IFNG^-/-^) mice, followed by LM infection. IFNG^-/-^ mice without CD8 Tm cell transfer were highly susceptible to LM as demonstrated by over 10^8^ CFUs in both the spleen and the liver at 72h post infection (**Fig 6A and 6B**). Adoptive transfer of CD8 Tm cells significantly reduced spleen and liver bacterial CFU numbers by around 2 logs (**Fig 6A and 6B**). Adoptive transferred CD8 Tm cells from progesterone treated mice, however, reduced spleen and liver bacterial CFU numbers by around only 1 log (**Fig 6A and 6B**). CD8 Tm cells from progesterone and decitabine treated mice were nearly as protective as those from progesterone untreated mice (**Fig 6A and 6B**). Moreover, the protection of CD8 Tm cells from progesterone and decitabine treated mice was abolished by *in vivo* administration of IFN-γ neutralizing antibody (**Fig 6A and 6B**). Consistent to the phenotype observed in IFN-γ neutralized mice, adoptive transferred IFN-γ-deficient CD8 Tm cells generated in IFNG^-/-^ mice (IFN-γ^-/-^ Tm) failed to reduce LM bacterial burden (**Fig 6C and 6D**), further suggesting that such a protection is dependent on IFN-γ produced by antigen-non-specific CD8 Tm cells. These data demonstrate that progesterone impairs IFN-γ-mediated immune protective functions of antigen-non-specific CD8 Tm cells via DNA hypermethylation.

## Discussion

In this study we show that in pregnant women, increased serum progesterone levels are associated with decreased IFN-γ production of CD8 Tm cells which is dependent on IFN-γ gene hypermethylation. Pregnant mice are highly susceptible to LM infection. And there are IFN-γ gene hypermethylation and reduced IFN-γ production in antigen-non-specific CD8 Tm cells in both pregnant mice and progesterone-treated non-pregnant female mice early after LM infection. Moreover, LM antigen-non-specific CD8 Tm cells from progesterone-treated mice have reduced protection against LM after adoptive transfer to pregnant mice or IFNG^-/-^ mice, which is dependent on progesterone-induced IFN-γ gene hypermethylation and reduced IFN-γ production early after LM infection.

Host defense against LM infection depends primarily on cellular immune responses[15,41]. The requirement of T cells, in particular CD8 T cells, in adaptive immune protection against intracellular pathogens such as LM has been well established in previous studies[41]. In mouse models, it has been shown that primary LM infection induces potent antigen-specific CD8 T cell immune responses that subsequently generates long-lasting antigen-specific CD8 Tm cells with augmented protective functions during secondary LM infection[42,43]. However, key molecules that are required for antigen-specific CD8 T cell-mediated immune protection against LM vary in different experimental settings[44,45]. Naïve IFNG^-/-^ mice are highly susceptible to LM infection, suggesting critical dependence of IFN-γ in bacterial clearance [45]. However, IFNG^-/-^ LM antigen-specific CD8 Tm cells adoptive transferred into naïve wild type mice provide equal protection as IFNG+/- CD8 Tm cells, suggesting an IFN-γ-independent mechanism through which antigen-specific CD8 Tm cells exert bacterial clearance[44]. More recent studies showed that antigen-non-specific CD8 Tm cells provide immune protection against LM via an IFN-γ dependent but cognate antigen independent mechanism[26,27]. Furthermore, IFN-γ-mediated protection by antigen-non-specific CD8 Tm cells is superior to that by NK cells due to the preferential co-localization of CD8 Tm cells with LM and macrophages in target organs post infection[27]. Importantly, such an antigen-non-specific CD8 Tm cell-mediated IFN-γ-production better reflect immune responses to LM in adult human with much more LM antigen-non-specific than antigen-specific CD8 Tm cells due to a highly diversified TCR repertoire[35]. Thus, IFN-γ-production by antigen-non-specific CD8 Tm cells plays indispensable roles in optimized host defense against LM infection[15,26]. Here in this study we generate a LM infection model in pregnant mice that have pre-established LM antigen-non-specific CD8 Tm cells, in order to determine the impact of increased progesterone on antigen-non-specific immune protective functions of CD8 Tm cells. We believe our current model reflect a critical aspect of the immune scenario of LM infection in pregnant women, which is not reflected in previous LM infection models in either antigen-inexperienced pregnant mice or LM antigen-specific CD8 Tm models in which LM antigen-specific CD8 Tm cells dominant the CD8 Tm cell repertoire[19,44].

Pregnant women and animals have significantly increased susceptibility to a variety of intracellular pathogens including LM, to which CD8 T cell-mediated immune responses are critically required[5,15,18,46]. Such an increased susceptibility during pregnancy has been associated with significantly increased female sex hormones such as progesterone and estrogen[1,7,18,46]. Indeed, most cases of LM infection during pregnancy were reported during the third trimester, when serum progesterone and estrogen levels reach the highest levels[18,28]. Progesterone plays sophisticated roles in immune cell functions presumably via both directly binding to cognate receptors or potentially undefined receptors, and indirectly through intermediate cells or molecules[7]. Functions of essentially all major immune cells including CD8 T cells are subject to the modulation of progesterone[7]. It has been reported that progesterone reduces IFN-γ-production by human CD8 T cells that express progesterone receptor[6], although the intracellular and molecular mechanisms remain unknown. By using human CD8 Tm cells from pregnant women, we show here that reduced IFN-γ-production by human CD8 Tm cells during pregnancy is dependent on IFN-γ gene hypermethylation. By using pregnant mice and female mice administered with exogenous progesterone at a dose level that reflect progesterone level in pregnant mice[38,39], we identify the causal relationship between increased progesterone and IFN-γ gene hypermethylation which is required to the reduced IFN-γ-production by antigen-non-specific CD8 Tm cells post LM infection. More importantly, the functional significance of progesterone-induced IFN-γ reduction in antigen-non-specific CD8 Tm cells is established, as demonstrated by the impaired anti-LM protection of antigen-non-specific CD8 Tm cells from progesterone treated mice in a hypermethylation-dependent manner. In contrast to the significant differences in LM bacterial burdens, we observed moderate but not statistically significant improvement in the number of viable fetuses and abortion rate in pregnant mice receiving antigen-non-specific CD8 Tm cells compared to those receiving no cell transfer or receiving CD8 Tm cells from progesterone treated mice. This might be explained by that IFN-γ production by antigen-non-specific CD8 Tm cells following LM infection disrupts maternal-fetal tolerance mechanisms, partially compensating the fetus-protective effects of reduced maternal and possibly placental bacterial burden[47,48]. Further mechanistic studies are critically required to extend our knowledge on whether and how maternal immune responses against LM infection during pregnancy may independently impact pregnancy outcomes.

It has been suggested that progesterone induces regulatory T cell expansion during pregnancy, which facilitates maternal-fetal tolerance but impairs anti-infectious immunity[47]. Earlier studies also suggested that placental trophoblasts as a protected niche to harbor bacteria that then re-seed maternal organs, causing persistent LM infection until expulsion of the infected placental tissues[48]. Our data thus provide a new mechanistic explanation to the T cell immune suppression and increased susceptibility to LM during pregnancy, which may work simultaneously and/or sequentially with the previously proposed mechanisms[47,48]. As CD8 Tm cell-derived IFN-γ is also required in innate-like protection against local virus infections[49], it remains possible that such an epigenetic modification of CD8 Tm cells by progesterone also contributes to the increased susceptibility to viral infections during pregnancy.

Transcription of IFN-γ gene is regulated by a variety of sequential epigenetic mechanisms including DNA methylation, transcription factors and chromatin modulation[29–32]. It has been shown in human T cells that hypermethylation of IFN-γ gene promoter CpG sites are related to immune suppression[50]. In mice, IFN-γ gene promoter CpG sites are nearly completely demethylated in effector CD8 T cells that are readily producing IFN-γ[31]. Although CD8 T m cells and naïve CD8 T cells have comparable overall IFN-γ gene methylation levels, quick demethylation of IFN-γ gene was observed in CD8 Tm cells but not naïve CD8 T cells early upon cognate antigen stimulation[31]. These findings strongly support an idea that DNA methylation is a gate-keeping molecular mechanism in controlling IFN-γ gene transcription in CD8 T cells including CD8 Tm cells. Here we show that hypermethylation of IFN-γ gene is associated with increased serum progesterone levels in pregnant women. Furthermore, hypermethylation of IFN-γ gene promoter region reduces IFN-γ production by CD8 Tm cells in pregnant women. Such a phenotype of hypermethylation-dependent IFN-γ reduction is also observed in CD8 Tm cells in pregnant mice. The hypermethylation of representative regulatory elements within the IFN-γ gene locus in CD8 Tm cells in pregnant mice further support our conclusions that progesterone induces hypermethylation-dependent IFN-γ reduction in CD8 Tm cells[51]. We also show that exogenous progesterone supplementation in non-pregnant female mice induces similar IFN-γ gene hypermethylation and IFN-γ reduction in CD8 Tm cells as observed in pregnant mice. These data demonstrate the indispensable roles of progesterone in CD8 Tm cell functions during pregnancy, although this does not exclude the potential impacts of other pregnancy-associated hormones on CD8 Tm cells. In a previous study, a rapid and nearly complete demethylation of IFN-γ gene was observed in CD8 Tm cells upon cognate antigen stimulation[31]. In our current model however, only partial demethylation was observed in antigen-non-specific CD8 Tm cells within 72h post LM infection. Such a partial demethylation is consistent to a relatively low frequency of IFN-γ-producing antigen-non-specific CD8 Tm cells upon cytokine stimulation *ex vivo* or LM infection *in vivo* in our current model. These findings indicate that DNA methylation-based regulation of IFN-γ gene transcription may have different upper stream signaling pathways when CD8 Tm cells are activated by TCR-dependent versus TCR-independent stimulants[52].

In conclusion, our data demonstrate that increased progesterone during pregnancy induces IFN-γ gene hypermethylation in CD8 Tm cells, resulting in reduced IFN-γ production and impaired anti-LM immune protective functions of antigen-non-specific CD8 Tm cells. These findings thus provide new mechanistic insights into the increased susceptibility to intracellular pathogens during pregnancy, as well as an unappreciated immune regulatory role of progesterone in CD8 Tm cells.

## Materials and methods

### Ethics statement

All experimental animal manipulations were conducted in accordance with the National Institute of Health Guide for the Care and Use of Laboratory Animals along with approval from the Scientific Investigation Board of Chinese PLA General Hospital. The project license number is NSFC81441006.

Written informed consent was obtained from all subjects for the use of personal medical data and peripheral blood cells in the current study. This study was conducted according to the Declaration of Helsinki and all procedures involving human subjects were approved by the Ethics Committee of the General Hospital of Chinese People's Armed Police Forces.

### Human CD8 Tm cell purification

Peripheral blood was collected at before, weeks 14 and 28 of pregnancy, and around 1 year after delivery from subjects who had prenatal exams in the Obstetric Out-Patient Clinic of the General Hospital of Chinese People's Armed Police Forces. At each time point, routine clinical examination on serum concentration of progesterone was performed in the Department of Clinical Laboratory Examinations of the General Hospital of Chinese People's Armed Police Forces. Peripheral blood mononuclear cells (PBMCs) were obtained by Ficoll-Paque gradient centrifuge[53].

CD8 Tm cells were negatively selected from non-adherent PBMCs by using human memory CD8 T cell enrichment kit (StemCell; Vancouver, BC, Canada). Purity of enriched human CD8 T memory cells was typically 90% to 95%, as determined by flow cytometry analysis on a CD3^+^CD8^+^CD45RO^+^ phenotype.

### Enzyme-Linked ImmunoSpot assay (ELISpot)

T2 (HLA-A0201^+^ TAP-deficient lymphoblastoid cell line) cells were kindly provided by Professor Xuetao Cao from Chinese Academy of Medical Sciences, and were cultured in RPMI 1640 culture media(Hyclone Laboratories; South Logan, UT, USA) supplemented with 10% fetal bovine serum (FBS; Hyclone Laboratories; South Logan, UT, USA), penicillin and streptomycin, at 37°C in a CO_2_ incubator. Human IFN-γ ELISpot kit was purchased from DAKEWE Biotech (Shenzhen, China). For T-cell receptor (TCR)-independent IFN-γ production, enriched CD8 Tm cells were cultured at 5×10^3^ cells/well in Serum-Free Media for ELISpot (DAKEWE; Shenzhen, China) supplemented with phytohaemagglutinin (PHA, 2.5μg/ml). For TCR-dependent IFN-γ production, enriched PBMC CD8 Tm cells from HLA-A0201^+^ donors were cultured at 2×10^4^ cells/well with T2 cells (CD8Tm:T2 = 10:1) pulsed with cytomegalovirus (CMV) pp65 peptide 495-503(NLVPMVATV, CMV_pp65_) at 10mg/ml. For demethylation treatment, enriched CD8 Tm cells were pretreated with decitabine (0.5μM; Xian-Janssen Pharmaceuticals Ltd, Xi’an, China) for 24 hours and the same concentration of decitabine was supplemented to the *ex vivo* stimulation system[53]. Cells were cultured in triplicate ELISpot wells for 16 hours according to manufacturer’s instructions and plates were read by DAKEWE Biotech (Shenzhen, China) on a AID EliSpot Read Classic (AID GmbH, Strassberg, Germany).

### Mice

Wild-type (WT) C57BL/6J (female and male, 6–8 weeks of age) were purchased from the Joint Ventures Sipper BK Experimental Animal Co. Ltd. (Shanghai, China). Breeders of Interferon-γ deficient (IFNG^-/-^) mice (B6.129S7-*Ifng*^*tm1Ts*^/J) were purchased from the Jackson Laboratory (Bar Harbor, ME, USA). Female IFNG^-/-^ mice at 6 to 12 weeks of age were used in the experiments. Mice were housed in specific pathogen-free conditions in central animal facility of Chinese PLA General Hospital.

### Immunization and infection of mice

Recombinant replication-deficient human type 5 adenovirus expressing ovalbumin (rAdHuOVA) was constructed and kindly provided by Dr. Xiaohua Tan from Beijing Military General Hospital. Female WT C57BL/6J mice or IFNG^-/-^ mice were immunized intramuscularly with rAdHuOVA at 5×10^7^ PFUs/animal. In the experiments where indicated, mice were immunized i.p. with 10^8.5^ egg infective dose units of H1N1 influenza A virus (A/PR8/34 strain; originally from ATCC and was a kind gift from Dr. Xiaohua Tan from Beijing Military General Hospital) to generate memory CD8 T cells[54–56]. At 40 days post immunization, OVA or influenza virus A (IVA) antigen-specific CD8 Tm cells were generated. And at 40 days post immunization, female mice with OVA antigen-specific CD8 Tm cells were mated with male C57BL/6J mice to induce pregnancy or leave non-pregnant. Female mice at 14 to 16 days of pregnancy were infected with LM. To determine the impact of progesterone on CD8 Tm cells, immunized non-pregnant female mice (on day 40 post immunization) were injected subcutaneously with progesterone (0.75mg/animal/day on days 1–7, and 1.5mg/animal/day on days 8–14; Sigma-Aldrich, St. Louis, MO, USA) suspended in 0.1ml olive oil or vehicle (0.1ml olive oil) for 14 consecutive days[8,57]. At various time points following progesterone administration, serum concentration of progesterone (P4) analysis was performed in the Department of Clinical Laboratory Examinations of the General Hospital of Chinese People's Armed Police Forces. In some progesterone treated mice, decitabine (1mg/kg/day) diluted in PBS was injected intraperitoneally on days 10–14 of progesterone administration[58].

A virulent strain of *Listeria monocytogenes* (LM; strain 10403S) was grown in brain-heart infusion broth (BHI; BD Biosciences, San Jose, CA, USA). At mid-log growth phase, colony forming units (CFUs) were counted following overnight incubation on BHI agar. For bacterial infection, mice were intravenously infected with LM diluted in PBS at 2.5×10^3^ CFUs/animal intravenously. At 72 h after LM challenge, spleen and liver were harvested and dissociated on metal screens in 10 ml of PBS containing 0.05% Triton-X100. Serial dilutions were performed in the same buffer and plated on BHI agar plates. Colonies on plates were counted after overnight culture and CFUs per organ were calculated. To determine pregnancy outcomes following LM infection, pregnant mice at day 13–15 of pregnancy, with or without OVA antigen-specific CD8 Tm cell transfer, were infected with LM at 2.5×10^3^ CFUs/animal intravenously. Uteri were examined 4 days post infection for post-implantation scars that indicate aborted fetuses, as well as for viable fetuses. Abortion rate = number of aborted fetuses/(number of aborted + viable fetuses)×100% [59].

### Flow cytometry staining, analysis, and cell sorting

Unless otherwise specified, all reagents for flow cytometry were purchased from BD Biosciences (San Jose, CA, USA). For intracellular staining of human CD8Tm cells, enriched PBMC CD8 Tm cells were stimulated *ex vivo* in triplicate wells with PMA(100ng/ml) and Ionomycin(1μg/ml) for 5 hours in the presence of GolgiPlug, followed by staining with human CD45-AF488(Biolegend, San Diego, CA USA), anti-human CD3-PerCP-Cy5.5, anti-human CD8-PE-Cy7, and anti-human CD45RO-APC. Stained cells were then fixed, permeabilized and stained with anti-human IFN-γ-PE. For *ex vivo* mouse T cell stimulation, single cell suspension from peripheral blood, the spleen and MLN of rAdHuOVA immunized mice were cultured in the presence of recombinant murine IL-12 (5 ng/ml) and IL-18 (10 ng/ml; both cytokines were from Peprotech, Rocky Hill, NJ, USA) for 24 hours. Five hours before harvest, GolgiPlug was supplemented to culture media. For intracellular flow cytometry staining of mouse cells, single cell suspension of peripheral blood, the spleen and MLN from LM infected mice were cultured *ex vivo* for 5 hours in the presence of GolgiPlug and stained with anti-mouse CD3-V450, anti-mouse CD8-APC-Cy7, anti-mouse CD44-APC, anti-mouse NK1.1-AF700 and H2K^b^/OVA.SIINFEKL tetramer-PE (H2K^b^OVA_257-264_; NIH Tetramer Core Facility, Atlanta, GA, USA). Stained cells were then fixed, permeabilized and stained with anti-mouse IFN-γ-FITC. In selected experiments, cells were stained with anti-mouse CD3-V450, anti-mouse CD8-APC-Cy7, H2K^b^/OVA.SIINFEKL tetramer-PE, and anti-mouse NKG2D-APC. FACS stained cells were acquired on a LSR II cytometer (BD Biosciences, San Jose, CA, USA). FACS data were analyzed by using FlowJo software version 10 (TreeStar, Ashland, OR, USA).

For adoptive transfer of CD8 Tm cells, cells from the spleen and MLN of rAdHuOVA or influenza virus A/PR8/34 immunized mice were enriched by using mouse CD8 negative selection kit (StemCell; Vancouver, BC, Canada). Enriched CD8^+^ cells were stained with anti-CD3, anti-CD8, anti-CD44 antibodies and H2K^b^OVA_257-264_ tetramer for OVA antigen specific CD8 Tm cells or H2D^b^NP366-374 –PE (NIH Tetramer Core Facility, Atlanta, GA, USA) for IVA antigen-specific CD8 Tm cells [54], followed by flow sorting on a Moflo XDP cell sorter (Beckman-Coulter; Brea, CA, USA) based on a CD3^+^CD8^+^CD44^+^tetramer^+^ phenotype. Purity of sorted CD8 Tm cells was >95%. Viability of purified CD8 Tm cells were >97% in all groups as determined by trypan blue exclusion. OVA or IVA antigen-specific CD8 Tm cells were adoptively transferred into recipient mice intravenously at 2×10^6^ cells/animal. Recipient mice were infected with LM (2.5×10^3^ CFUs/animal) 4 hour after CD8 Tm cell adoptive transfer. At 24 hours post infection, the presence of adoptively transferred OVA antigen-specific CD8 Tm cells and their IFN-γ-producing capacity were determined by flow cytometry analysis. To block IFN-γ *in vivo* in IFNG^-/-^ mice adoptively transferred with CD8 Tm cells, mice were injected intraperitoneally with 200 μg/animal of the anti-IFN-γ antibody (BioxCell, West Lebanon, NH, USA) on day -1 of infection. Dose of anti-IFN-γ antibody was repeated at 100 μg/animal/day on days 0, 1, and 2 of infection.

### Quantitative RT-PCR analysis

Total RNA was extracted from enriched human PBMC CD8 Tm cells(hCD3^+^hCD8^+^hCD45RO^+^) using miRNeasy Mini Kit (Qiagen, Germantown, MD, USA) according to manufacturer’s instruction. RT was performed using Reverse Transcription System (Promega; Madison, WI, USA) on 1 μg of total RNA[60]. Expression of IFN-γ and GAPDH was quantified by SYBRgreen real-time quantitative PCR analysis on an Mx3000p light cycler (Stratagene; La Jolla, CA, USA), and data were analyzed using Mx3000p software (Stratagene; La Jolla, CA, USA). Primers for human IFN-γ (forward and reverse): 5’-GCAGGTCATTCAGATGTAGCGG-3’ and 5’-TGTCTTCCTTGATGGTCTCCACAC-3’. Primers for human GAPDH (forward and reverse): 5’-GAGTCAACGGATTTGGTCGT-3’ and 5’-TTGATTTTGGAGGGATCTCG-3’. IFN-γ mRNA expression was expressed as 2^-ΔCT^ relative to GAPDH.

### Bisulfite sequencing

Genomic DNA was prepared from purified human PBMC CD8 Tm cells(hCD3^+^hCD8^+^hCD45RO^+^), or murine splenic CD8 Tm cells (CD3^+^CD8^+^CD44^+^H2K^b^-OVA_257-264_ tetramer^+^) at 40 days post immunization, by using the Wizard Genomic DNA Purification Kit (Promega; Madison, WI, USA). Bisulfite-treatment of genomic DNA was performed as previously described[60], followed by PCR amplification using the Epitectbisulfit kit (Qiagen; Germantown, MD, USA). For methylation analysis on human IFN-γ gene promoter CpG sites, the following primer pair was used: forward, 5’-TGTGAATGAAGAGTTAATATTTTATTA-3’; reverse, 5’-TTGGTAGTAATAGTTAAGAGAATTTA-3’[50]. For methylation analysis on mouse IFN-γ gene promoter CpG sites, bisulfite-treated DNA was amplified in semi-nested PCR using primers: 5’-GGTGTGAAGTAAAAGTGTTTTTAGAGAATTTTAT-3’ and 5’-CAATAACAACCAAAAACAACCATAAAAAAAAACT-3’, then 5’-GGTGTGAAGTAAAAGTGTTTTTAGAGAATTTTAT-3’ and 5’-CCATAAAAAAAAACTACAAAACCAAAATACAATA-3’[33]. For methylation analysis on regulatory elements of mouse IFN-γ gene locus, the following primers were used: Locus -54: Primer pair 1, 5’- GTGGTTAAGATAGGTTTGTTAGTGGTTTGTT-3’, 5’- ATTACACATCTACATAATCTAAAAACTTCCTA-3’; Primer pair 2, 5’- GGTTTGTGGATATTAGTGATGTTGAG-3’, 5’- AAACACTTCCTTCAACTTCCCCTACTATA-3’. Locus -6: 5’- TTTAATTTATGGGATAAATGAGTTA-3’, 5’- AAATACTATCACCCCAATAACACATC-3’. Locus +18: 5’- TAATGTGAGTTGGAATATTAAGAATTT-3’, 5’- TCTAAATAAACAAATCACCAAATCTCA-3’. Locus +20: 5’- GATAAGTAGTTTAAAGGTTATATGT-3’, 5’- CTAAATCCCTTACTAACCTACATCC-3’. Locus +55: Primer pair 1, 5’- GAAGGTTTTATGTTTAGGTTAGAAATATTTT-3’, 5’- TACCTATCTCTTACCCAAAATATTATCTATA-3’; Primer pair 2, 5’- GATGTTTGGAGAGAGATAAAATATAGGTTAGTT-3’, 5’- TTTCCTACAAATAATTCTCTAATTA-3’[51]. The PCR products were gel purified and cloned into the pGEM-T vector (Promega; Madison, WI, USA). The inserted PCR fragments of individual clones were sequenced using an ABI PRISMDNA sequencer (Applied Biosystems; Foster City, CA, USA). For all samples, 10 reads or 6 reads per CpG site were used to determine the average percentage of methylated CpG.

### Statistical analysis

Two-tailed unpaired Student’s *t*-test was used for statistical comparison between two groups in mouse experiments. Two-tailed paired Student’s *t*-test was used for statistical comparison between control and decitabine treatment groups in human cell experiments. One-way ANOVA and Tukey’s multiple comparisons test was used to compare between multiple groups. Pearson correlation analysis was used to determine the potential correlation between two parameters. All statistical analysis was performed by using the GraphPad Prism software (version 6.01; GraphPad Software, La Jolla, CA, USA). Values of P < 0.05 were considered statistically significant.

## Supporting information

S1 Fig Relative IFN-γ mRNA expression during pregnancy. (TIF)

S2 Fig Generation of OVA-specific CD8 Tm cells in pregnant mice. (TIF)

S3 Fig IFN-γ gene methylation in CD8 Tm cells in pregnant mice. (TIF)

S4 Fig Expression of NKG2D in CD8 Tm cells in pregnant mice. (TIF)

S5 Fig Serum concentration of progesterone in non-pregnant female mice injected with progesterone. (TIF)

S6 Fig IFN-γ production in H2K^b^OVA tetramer^-^ CD8 T cells and NK cells. (TIF)

S7 Fig Progesterone-induced IFN-γ gene methylation in CD8 Tm cells. (TIF)

S8 Fig IFN-γ production by adoptive transferred OVA antigen-specific CD8 Tm cells. (TIF)

S9 Fig Pregnancy outcomes in LM-infected mice receiving progesterone-treated CD8 Tm cells. (TIF)
