# Supplementary figures and images for: Progesterone impairs antigen-non-specific immune protection by CD8 T memory cells via interferon-γ gene hypermethylation
Source: PLoS Pathog. 2017 Nov 20;13(11):e1006736. doi: 10.1371/journal.ppat.1006736 (PMC5714395; doi:10.1371/journal.ppat.1006736)

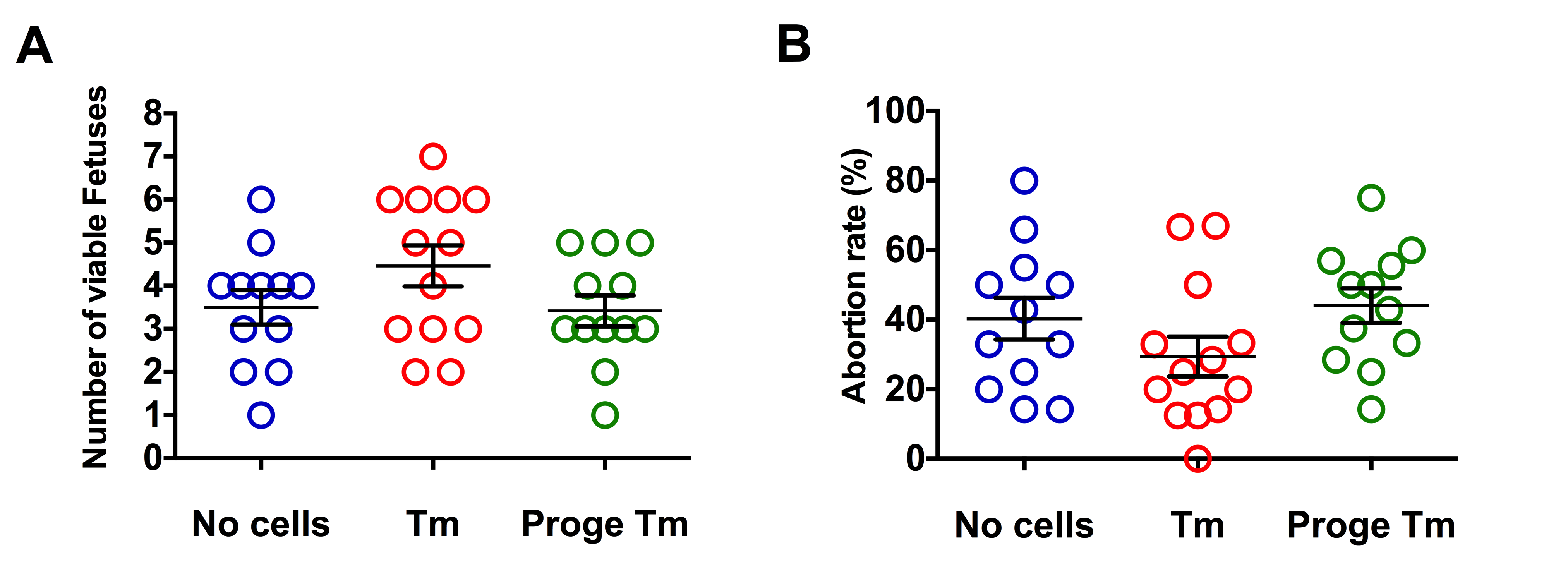

Supplement: S9 Fig — (TIF) [file ppat.1006736.s009.tif]
